# Research Situation, Hotspots, and Global Trends in Laser Treatment of Acne Scars: A Bibliometric Analysis of Related Research From 2014 to 2024

**DOI:** 10.1111/jocd.16663

**Published:** 2024-11-11

**Authors:** Yang Wen, Yuan Cai, Lanfang Zhang, Lin Li, Jing Wang, Feng Jiang, Nana Sun, Ni Zeng

**Affiliations:** ^1^ Department of Dermatology, Guizhou Province Cosmetic Plastic Surgery Hospital Affiliated Hospital of Zunyi Medical University Zunyi China; ^2^ Department of Dermatology Affiliated Hospital of Zunyi Medical University Zunyi China; ^3^ Department of Neonatology Obstetrics and Gynecology Hospital of Fudan University Shanghai China

**Keywords:** acne scar, bibliometrics, citespace, laser, VOSviewer

## Abstract

**Background:**

Acne vulgaris is a chronic inflammatory skin condition, commonly resulting in acne scars. Treating acne scars remains a significant challenge in dermatology. With advancements in laser technology, its clinical use for treating acne scars has been increasing annually. However, bibliometric analysis on laser treatment for acne scars is lacking. This study aims to use bibliometrics to comprehensively understand the development trends and research hotspots in laser treatment for acne scars.

**Methods:**

Using “acne scar” and “laser” as search terms, literature on laser treatment of acne scars from 2014 to 2024 was retrieved from the Web of Science Core Collection (WoSCC) database. The literature data were visualized using VOSviewers, CiteSpace, and R software, generating maps of countries, research institutions, authors, journals, references, and keywords.

**Results:**

The analysis included 536 articles from 46 countries, with the United States and China leading in publications. Publications focusing on laser treatment of acne scars exhibit a consistent growth trend annually. Key research institutions include Mahidol University in Thailand, Cairo University in Egypt, and Hallym University in South Korea. The “Journal of Cosmetic Dermatology” had the highest number of articles in this field, while “Dermatologic Surgery” was the most cited publication. These publications involved contributions from 2135 authors, with Professor Manuskiatti Woraphong from Mahidol University in Thailand being the most prolific author in this field. Research on the efficacy of laser treatment for acne scars is a major focus in this field. Among the different types of lasers, CO_2_ lasers are the most commonly used. Emerging research focuses include therapies such as platelet‐rich plasma, picosecond laser, trichloroacetic acid, and burn scar.

**Conclusions:**

Treatment efficacy is the primary focus of research in the field of laser treatment for acne scars. Platelet‐rich plasma, and novel picosecond lasers, have emerged as hot topics and trends in this research field. However, it is important to note that the impact factors of journals publishing in this field are currently low. Therefore, clinicians must consider and explore strategies for publishing high‐quality clinical research in the future.

## 1 Introduction

Acne vulgaris is a chronic inflammatory skin disease affecting the pilosebaceous unit, caused by hypersecretion of androgens, increased sebum production, follicular hyperkeratinization, and infection by *Propionibacterium acnes*. It commonly manifests on the face, chest, upper arms, and back, characterized by comedones, inflammatory papules, cysts, nodules, and pustules [1, 2, 3]. Statistics indicate that acne affects approximately 80% of adolescents, with a global prevalence of 9.4%, making it the eighth most common disease worldwide [1]. Improper handling, delayed diagnosis and treatment, and severe acne contribute to the development of acne scars. The incidence of acne scars ranges from 1% to 11% [4]. Adolescents with acne scars may experience low self‐esteem and, in severe cases, may develop psychological issues such as anxiety and depression [5].

The etiology of acne scars is unclear, and the mechanisms vary among different scar types. Factors such as *Cutibacterium acnes (C. acnes)*, *Propionibacterium acnes* infection, abnormal immune responses, cytokine imbalances, and genetic predispositions contribute to collagen synthesis and degradation abnormalities, leading to acne scars [6]. Excessive collagen deposition results in hypertrophic acne scars and keloids, while decreased collagen deposition leads to atrophic scars [7, 8]. Common treatments for acne scars include surgical interventions, laser therapy, radiofrequency treatments, chemical peels, and emerging modalities like adipose tissue transplantation, platelet‐rich plasma therapy (PRP), and hyaluronic acid‐based fillers [9]. Although these treatments can improve scars, each modality has limitations. Treating acne scars remains a clinical challenge. Lasers are often used as alternative treatments or in combination therapies, showing promising efficacy [10, 11]. Laser therapy in clinical practice is classified by different criteria. Lasers are categorized as ablative or nonablative based on epidermal integrity. Ablative lasers include carbon dioxide (CO_2_ laser) and erbium‐doped yttrium aluminum garnet (Er: YAG) lasers, while nonablative lasers include erbium‐doped glass, neodymium‐doped yttrium aluminum garnet (Nd: YAG), semiconductor lasers, and thulium lasers [12]. Based on mechanisms of action, lasers can be categorized into ablative fractional and nonablative fractional lasers. Both modalities induce thermal damage through selective photothermolysis and photobiostimulation, stimulating collagen remodeling and increasing elastic fiber density. The choice of laser or combination therapies in a staged approach depends on the patient's skin type and acne scar characteristics (depth, inflammation) [13]. Numerous laser modalities are available for treating acne scars, and considerable research on laser therapy for acne scars exists internationally. However, comprehensive data compilation in this research is relatively lacking.

Bibliometrics is a method for quantitative and qualitative analysis of the production and status of publications in specific research fields [14, 15]. It provides comprehensive insights into authors, keywords, journals, countries, institutions, and references. Researchers often use tools like VOSviewers, CiteSpace, and R packages to visualize bibliometric analysis results. These tools are widely used in the medical field. Many dermatologists have used bibliometric analysis to publish articles on hidradenitis suppurativa, rosacea, and psoriasis [16, 17, 18]. However, no visualized studies on laser therapy for acne scars have been reported. Therefore, this study proposes using bibliometric methods with the Web of Science Core Collection to collect research on laser applications for acne scars over the past decade (2014–2024). The aim was to summarize the growth trends of publications, authors, institutions, countries, and research hotspots in laser therapy for acne scars. These findings will provide valuable insights for topic selection, collaboration, and future development trends in this field.

## 2 Materials and Methods

### 2.1 Data Collection

**FIGURE 1:**
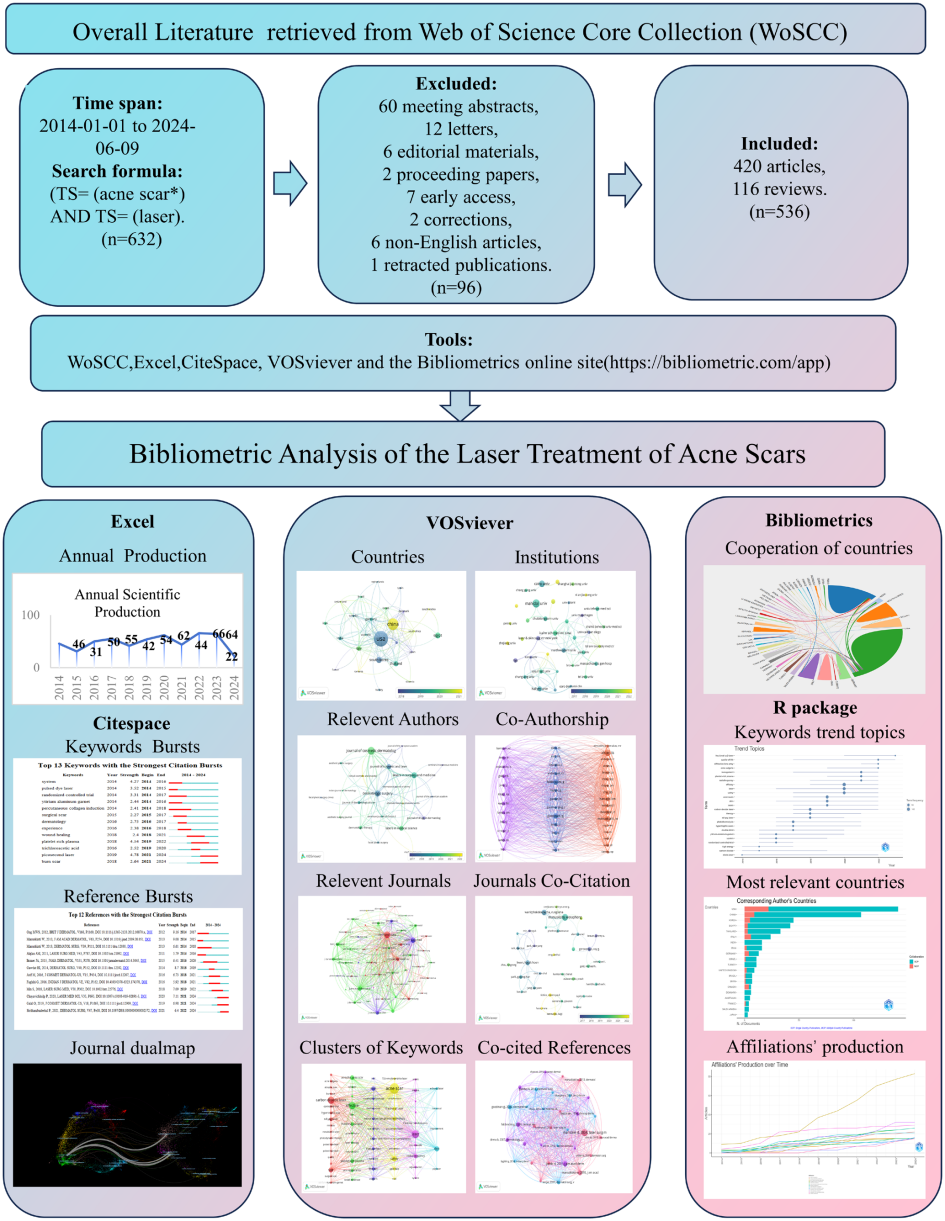
The workflow of this research for laser treatment of acne scars.

We searched the Web of Science Core Collection for literature published between 2014 and 2024 using “acne scar” and “laser” as keywords. We retrieved a total of 632 documents. After excluding 96 documents that did not meet the criteria (meeting abstracts, non‐English articles, letters, editorial materials, proceeding papers, early access, corrections, retracted publications), 536 documents were included for analysis. All information was downloaded on June 09, 2024, as shown in Figure 1.

### 2.2 Data Analysis and Visualization

Data on annual publications and citations are from WoSCC. The remaining information from 1143 manuscripts was converted into a text file and then imported into CiteSpace 6.1.R6 Advanced, VOSviewer 1.6.19, and the Bibliometric online site (https://bibliometric.com/app). CiteSpace is a Java‐based information visualization tool that analyzes the body of literature in a given field. It aims to explore key trends in the development of the field and generate visualizations that depict the field's frontiers and evolution. In this study, we used CiteSpace to visualize clustered analysis of co‐occurring keywords, the keywords with citation bursts, keywords with timelines, and clustered analysis of co‐citation references and journals co‐citation. The main parameters used were as follows: the time was sliced into 1‐year intervals from January 2000 to September 2023. The selection criteria for analysis were the g‐index and a scale factor of *k* = 25. We used this software to visualize the data, representing the number of articles published as the size of the nodes and showing collaborations between nodes with lines. The color of the lines indicates the first collaboration year, with purple representing collaborations from a long time ago and yellow representing more recent collaborations. VOSviewer 1.6.19 is a bibliometric analysis software frequently utilized for extracting key information from multiple publications. This software is commonly employed to construct collaboration, co‐citation, and co‐occurrence networks. We used VOSviewer to analyze co‐authorship, including countries, institutions, and authors, as well as keywords co‐occurrence, and journals. The size and color of the nodes indicate the number or rank of these elements, and the thickness of the lines connecting the nodes represents the level of collaboration or shared references within the project. Finally, we used the Bibliometric online site (https://bibliometric.com/app) to analyze the linkages between countries. Figure 1 illustrates the workflow of this study.

## 3 Results

### 3.1 Statistical Analysis of Publications

**FIGURE 2:**
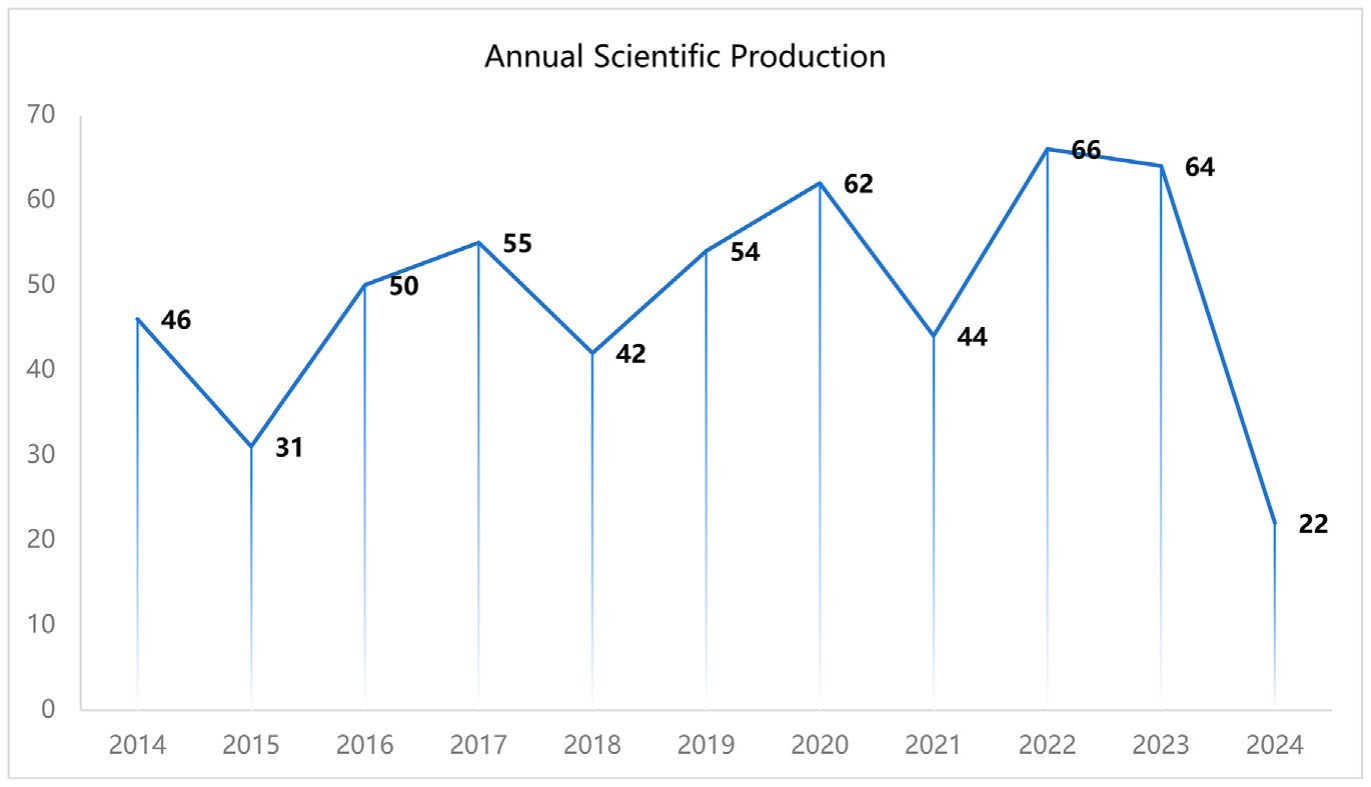
Trend of annual publication output in laser treatment for acne scars research field on web of science from 2014 to 2024.

A total of 536 documents from the Web of Science database were included after literature search and screening. Figure 2 shows the annual publication trend of laser treatment for acne scars from 2014 to 2024. Overall, the number of publications in this field has been increasing. The lowest number of publications was in 2015, with 31 documents. From 2016 onward, the number of publications gradually increased, peaking at 66 documents in 2022.

### 3.2 Country and Institutional Analysis

**FIGURE 3:**
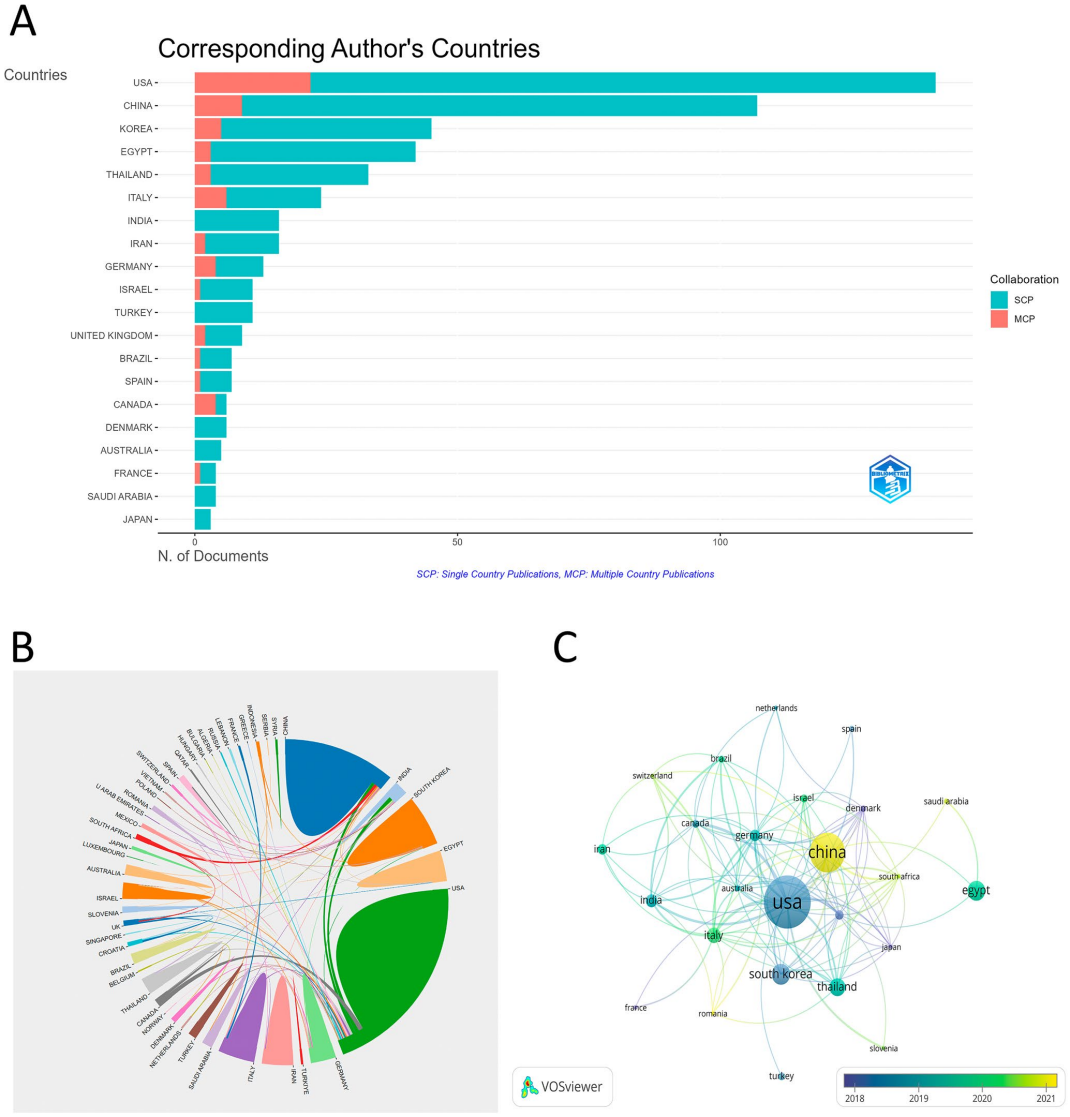
Visualization of leading countries in the field of laser treatment for acne scars. (A) Top 20 most relevant countries. (B) Cooperation of countries. (C) Visual analysis of countries.

Literature on laser treatment for acne scars originates from 50 countries and 833 institutions. The United States was at the top with single‐country publications, multicountry publications, and the highest citations and total link strength as shown in Figure 3A and Table 1. Furthermore, the United States, China, and South Korea lead the world regarding scientific productivity (Figure 3B). Then, we screened and visualized 25 countries with four or more publications, creating a collaboration network based on publication numbers and relationships (Figure 3C). Since 2020, countries like South Africa, China, and Italy have seen a significant increase in publication volume, as shown in Figure 3C.

**TABLE 1:** List of the top 10 countries and regions with the highest research productivity.

Rank	Countries	Counts	Citations	Total link strength
1	USA	170	3370	76
2	China	112	934	29
3	South Korea	46	818	16
4	Egypt	43	569	4
5	Thailand	37	596	18
6	Italy	30	363	36
7	India	23	425	24
8	Germany	19	369	34
9	Iran	17	157	6
10	The United Kingdom	15	254	25

**FIGURE 4:**
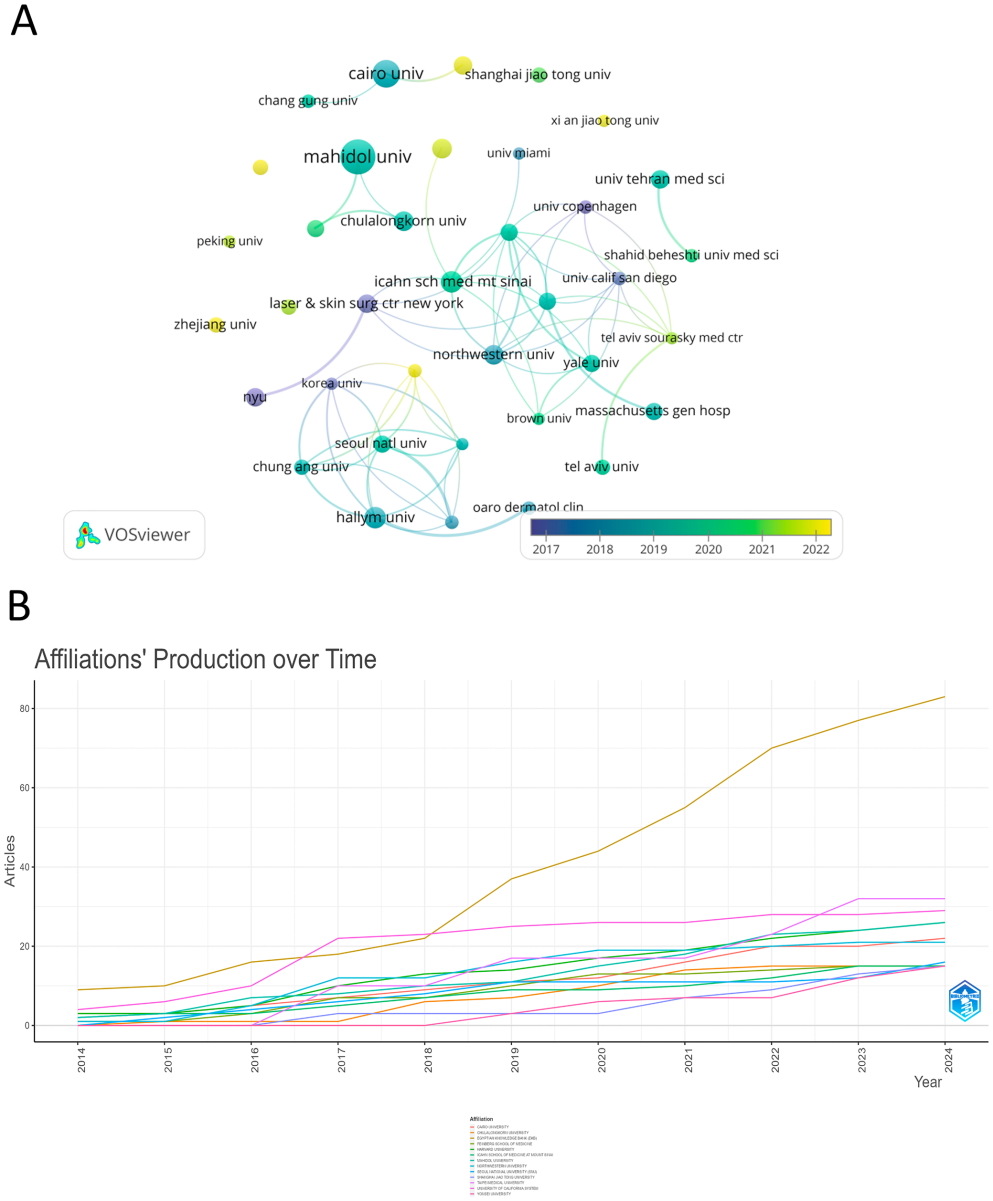
The visualization of institutions. (A) Visual analysis of institutions in laser treatment for acne scars research field. (B) The map of affiliations' production over time.

The top 10 institutions in laser treatment for acne scars are spread across five countries (Table 2). The three institutions with the most publications are Mahidol University, Thailand (*n* = 22), Cairo University, Egypt (*n* = 16), and *Hallym Univ*ersity, South Korea (*n* = 11). We then selected 37 institutions with at least five publications for visualization, constructing a collaboration network based on publication numbers and relationships (Figure 4A). Figure 4A highlights the close collaboration between Northwestern University, New York University, the New York Laser and Skin Surgery Center, Harvard Medical School, and the University of California, San Diego. Additionally, there is active collaboration among three Thai universities: Mahidol University, Chulalongkorn University, and Thammasat University. It is worth noting that, the publications of the journals of the Egyptian knowledge bank are growing rapidly (Figure 4B).

**TABLE 2:** Top 10 institutions in laser treatment for acne scars research field from 2014 to 2024.

Rank	Institutions	Counts	Citations
1	Mahidol University (Thailand)	22	418
2	Cairo University (Egypt)	16	304
3	Hallym University (South Korea)	11	242
4	Icahn School of Medicine at Mount Sinai (American)	11	175
5	Chulalongkorn University (Thailand)	10	99
6	University Magna Græcia of Catanzaro (Italy)	10	91
7	Northwestern University (American)	10	150
8	Al Azhar University (Egypt)	9	47
9	Laser & Skin Surgery Center of New York (American)	9	377
10	New York University (American)	9	416

### 3.3 Journals and Co‐Cited Publications

**FIGURE 5:**
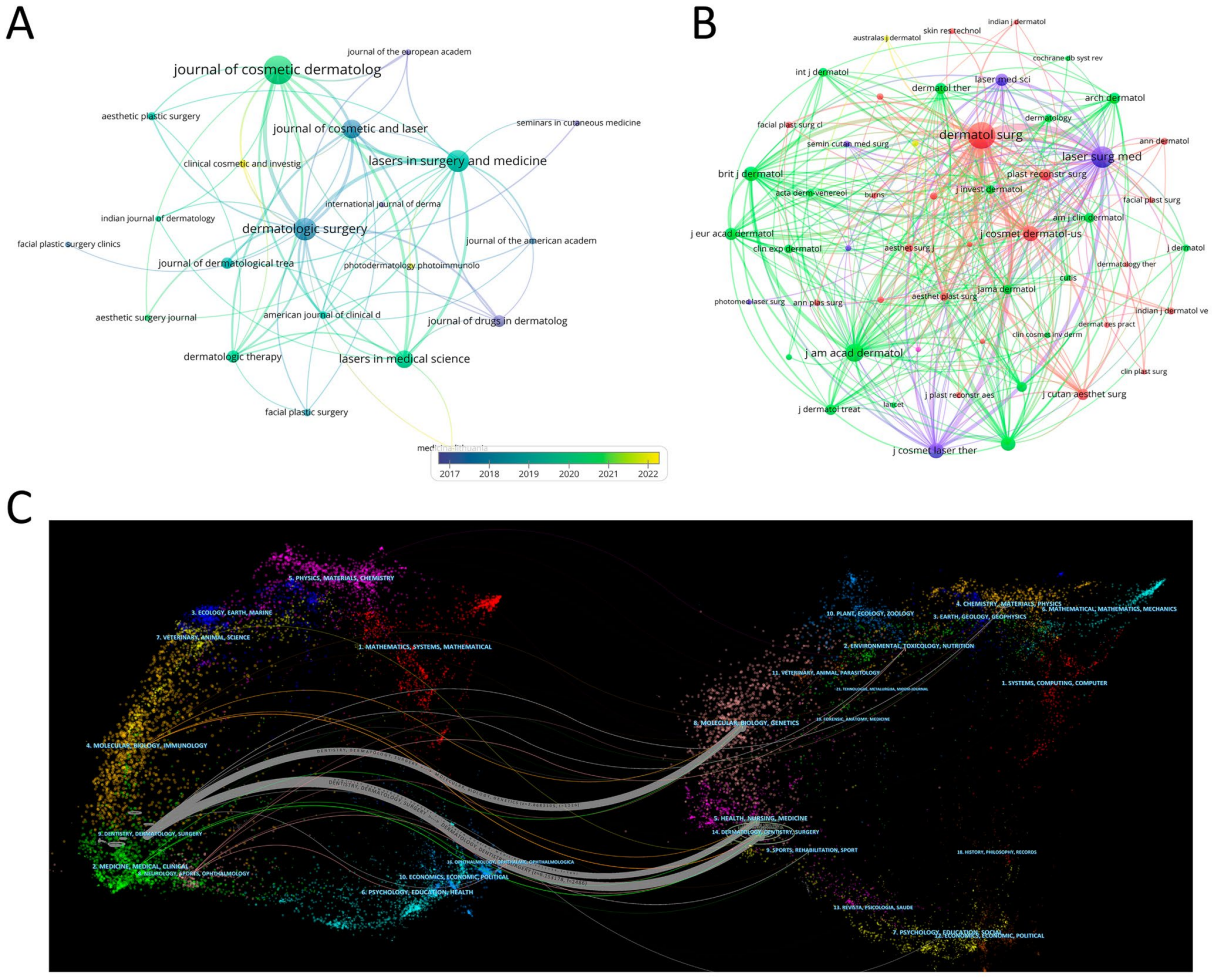
The journals analysis. (A) Visual analysis of journals in laser treatment for acne scars research field. (B) Visual analysis of co‐cited journals in laser treatment for acne scars research field. (C) The journal dualmap overlay showcases the interconnections among various journals in the field of laser treatment for acne scars.

Literature on laser treatment for acne scars is distributed across 105 journals. The Journal of Cosmetic Dermatology has the highest number of publications (*n* = 79), followed by Dermatologic Surgery (*n* = 45), Lasers in Surgery and Medicine (*n* = 50), and the Journal of Cosmetic and Laser Therapy (*n* = 39) (Table 3). Among the top 12 journals, the Journal of the American Journal of Clinical Dermatology has the highest impact factor (IF = 7.3), followed by Dermatologic Therapy (IF = 3.6). We then selected journals with at least five related publications for visualization, creating a journal network (Figure 5A). Figure 5A shows that Dermatologic Surgery has active citation relationships with Lasers in Surgery and Medicine, the Journal of Cosmetic and Laser Therapy, and the Journal of Cosmetic Dermatology.

**TABLE 3:** Top 12 journals in laser treatment for acne scars research and co‐cited journals.

Rank	Journals	Counts	IF	*Q*	Co‐cited Journals	Co‐citations	IF	*Q*
1	*Journal of Cosmetic Dermatology*	79	2.3	Q4	*Dermatologic Surgery*	2912	2.914	Q3
2	*Dermatologic Surgery*	52	2.914	Q2	*Journal of the American Academy of Dermatology*	1077	13.8	Q1
3	*Lasers in Surgery and Medicine*	50	2.4	Q2	*Journal of Cosmetic Dermatology*	753	2.3	Q4
4	*Journal of Cosmetic and Laser Therapy*	39	1.2	Q4	*Journal of Cosmetic and Laser Therapy*	737	1.2	Q4
5	*Lasers in Medical Science*	35	2.1	—	*British Journal of Dermatology*	626	11.113	Q1
6	*Dermatologic Therapy*	20	3.6	Q4	*Veterinary Dermatology*	341	1.4	—
7	*Journal of Drugs in Dermatology*	18	1.5	Q4	*Dermatologic Therapy*	320	3.6	Q4
8	*Journal of Dermatological Treatment*	17	3.23	Q4	*American Journal of Clinical Dermatology*	269	7.3	Q2
9	*Aesthetic Plastic Surgery*	10	2.708	Q4	*Journal of Clinical and Aesthetic Dermatology*	255	—	—
10	*Clinical Cosmetic and Investigational Dermatology*	10	2.3	Q4	*International Journal of Dermatology*	203	3.6	Q4
11	*American Journal of Clinical Dermatology*	9	7.3	Q2	*Clinical and Experimental Dermatology*	186	4.1	Q4
12	*Facial Plastic Surgery*	9	1.0	Q1	*Aesthetic Plastic Surgery*	165	2.708	Q4

As shown in Table 3, among the 12 most co‐cited journals, two have been cited over 1000 times. Dermatologic Surgery (co‐citations = 2912) is the most frequently cited, followed by the Journal of the American Academy of Dermatology (co‐citations = 1077), the Journal of Cosmetic Dermatology (co‐citations = 753), and the Journal of Cosmetic and Laser Therapy (co‐citations = 737). The Journal of the American Academy of Dermatology has the highest impact factor (IF = 13.8), followed by the British Journal of Dermatology (IF = 11.113). We selected journals with at least 50 co‐citations for visualization analysis, as shown in Figure 5B. This figure illustrates the co‐citation relationships between Dermatologic Surgery and Lasers in Surgery and Medicine, the Journal of Cosmetic and Laser Therapy, and the Journal of the American Academy of Dermatology.

The dual‐map overlay of journals shows the citation relationships between journals and co‐cited journals, with clusters of citing journals on the left and clusters of cited journals on the right. As shown in Figure 5C, the gray path is the main citation path, which represents the research published in Dentistry/Dermatology/Surgery journals is mainly cited by literature in Molecular/Biology/Genetics journals, Health/Nursing/Medicine journals, and Dermatology/Dentistry/Surgery journals.

### 3.4 Analysis of Authorship and Co‐Cited Authors

**FIGURE 6:**
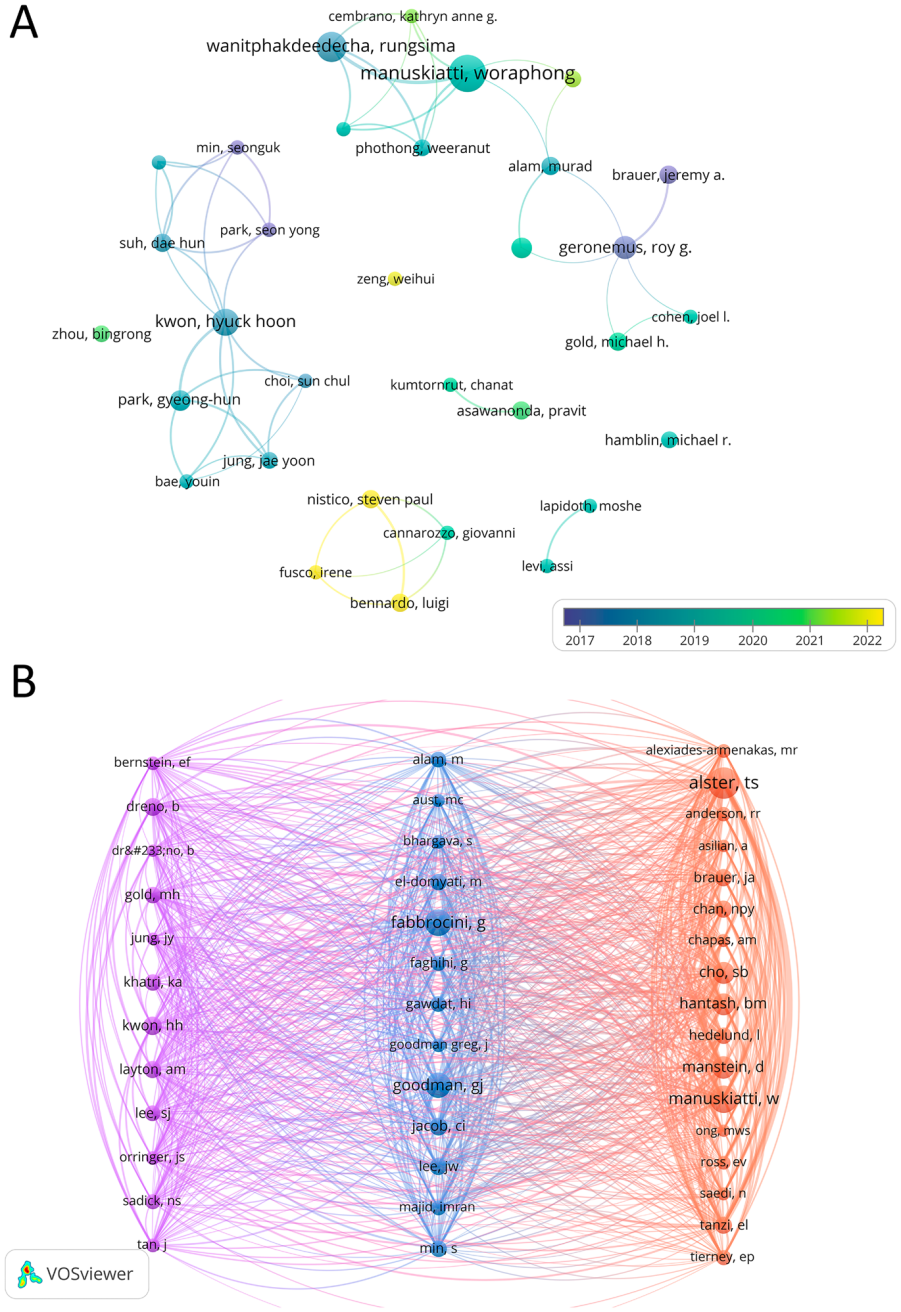
Visual analysis of authors (A) and co‐cited authors (B) in laser treatment for acne scars research field based on VOSviewer.

We conducted a visualization analysis of contributions from 2153 different authors in the research field of laser treatment for acne scars. The analysis revealed that Professor Manuskiatti Woraphong from Mahidol University, Thailand, has the most publications, with a total of 16 papers (Table 4), followed by Wanitphakdeedecha Rungsima with 12 papers. By analyzing authors with four or more papers, we constructed a collaboration network (Figure 6A), revealing extensive cooperation among researchers. For instance, Kwon Hyuck‐Hoon collaborates closely with Park Gyeong‐Hun, Geronemus Roy G, and Alam Murad.

**TABLE 4:** Top 10 authors and co‐cited authors list in laser treatment for acne scars.

Rank	Authors	Counts	Co‐cited authors	Co‐citations
1	Manuskiatti Woraphong	16	Alster Tina	191
2	Wanitphakdeedecha Rungsima	12	Manuskiatti Woraphong	152
3	Kwon Hyuck‐Hoon	10	Fabbrocini Gabriella	146
4	Geronemus Roy G	8	Goodman Greg J	134
5	Dover Jeffrey S	7	Manstein Dieter	110
6	Park Gyeong‐Hun	7	Hantash Basil M	103
7	Alam Murad	6	Cho Sung‐Baek	102
8	Asawanonda Pravit	6	Kwon, Hyuck‐Hoon	79
9	Bennardo Luigi	6	Jacob Carolyn I	76
10	Brauer Jeremy A	6	Brauer, Jeremy A	71

Among the 7807 co‐cited authors, seven have received more than 100 citations (Table 4). Alster Tina from Washington State University has the highest number of citations among co‐cited authors, with a total of 191 citations, followed by Manuskiatti Woraphong (*n* = 152), Fabbrocini Gabriella (*n* = 146), and Goodman Greg J (*n* = 134). We filtered authors with 100 or more co‐citations and created a co‐citation network for visualization analysis (Figure 6B). Notably, there are dynamic collaborations among frequently cited authors in the literature. For example, there are close connections between Alster Tina, Hantash Basil M, and Manuskiatti Woraphong, as well as among Fabbrocini Gabriella, Jacob Carolyn I, and Goodman Greg J.

### 3.5 Analysis of Co‐Cited References

**FIGURE 7:**
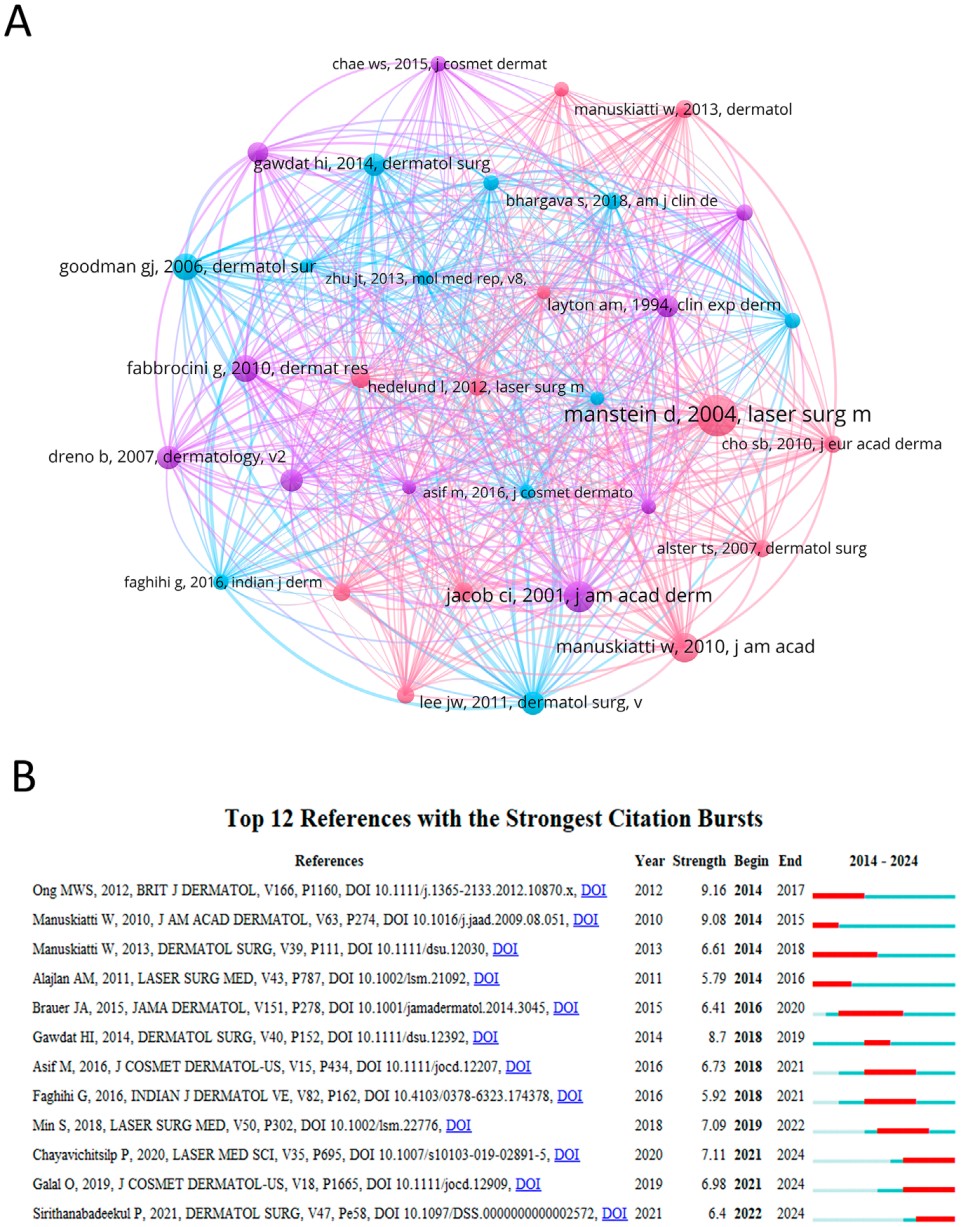
The references analysis. (A) Visual analysis of co‐cited references in laser treatment for acne scars based on VOSviewer. (B) Top 12 references with the strongest citation bursts based on CiteSpace involved in laser treatment for acne scars.

The phenomenon where two documents are cited together by another document is known as co‐citation. Over the past decade, there have been 10 778 co‐cited references in the field of laser treatment for acne scars. Among the top 10 co‐cited articles listed in the references (Table 5), each has been co‐cited at least 51 times, with three being cited more than 70 times. We selected references cited more than 40 times to construct a co‐citation network (Figure 7A). Figure 7A prominently highlights the active co‐citation relationships between references such as “Manstein D, 2004, Laser Surg Med, v34, p426, doi 10.1002/lsm.20048” and others like “Manuskiatti W, 2010, J Am Acad Dermatol, v63, p274, doi 10.1016/j.jaad.2009.08.051,” “Fabbrocini G, 2010, Dermatol Res Pract, v2010, doi 10.1155/2010/893080,” and “Jacob CI, 2001, J Am Acad Dermatol, v45, p109, doi 10.1067/mjd.2001.113451.”

**TABLE 5:** Top 10 co‐cited references in laser treatment for acne scars.

Rank	Co‐cited references	Co‐citations
1	Manstein d, 2004, laser surg med, v34, p426, doi 10.1002/lsm.20048	101
2	Jacob ci, 2001, j am acad dermatol, v45, p109, doi 10.1067/mjd.2001.113451	75
3	Manuskiatti w, 2010, j am acad dermatol, v63, p274, doi 10.1016/j.jaad.2009.08.051	70
4	Fabbrocini g, 2010, dermat res pract, v2010, doi 10.1155/2010/893080	63
5	Goodman gj, 2006, dermatol surg, v32, p1458, doi 10.1111/j.1524‐4725.2006.32354.x	62
6	Dreno b, 2007, dermatology, v214, p46, doi 10.1159/000096912	55
7	Lee jw, 2011, dermatol surg, v37, p931, doi 10.1111/j.1524‐4725.2011.01999.x	55
8	Brauer ja, 2015, jama dermatol, v151, p278, doi 10.1001/jamadermatol.2014.3045	52
9	Gawdat hi, 2014, dermatol surg, v40, p152, doi 10.1111/dsu.12392	52
10	Layton am, 1994, clin exp. dermatol, v19, p303, doi 10.1111/j.1365‐2230.1994.tb01200.x	51

Citation burst references are those frequently cited by scholars within a specific field over a defined period. In this study, 12 reference with strong citation bursts were identified by using CiteSpace (Figure 7B). As illustrated in Figure 7B, the red bars represents strong citation bursts. The citation bursts of references occurred as early as 2014 and as late as 2022. The reference with the strongest citation burst (strength = 9.16) is the article entitled “Fractional laser resurfacing for acne scars: a review,” authored by Ong MW et al., with citation burst occurred from 2014 to 2017. The reference with the second strongest citation burst (strength = 9.08) is titled “Efficacy and Safety of a Carbon‐Dioxide Ablative Fractional Resurfacing Device for the Treatment of Atrophic Acne Scars in Asians,” authored by Manuskiatti W et al., with citation burst occurred during the period from 2014 to 2015. Overall, the burst strength of the 12 reference ranged from 5.79 to 9.16, and endurance strength from 1 to 4 years. Table 6 presents a summary of the main research contents of these 12 reference, organized according to the sequence of the reference depicted in the figure.

**TABLE 6:** Twelve references to primary research with strong citation bursts.

Rank	Strength	Main research content
1	9.16	Investigates the effectiveness of ablative and nonablative fractional photothermolysis (FP) lasers for treating facial acne scars.
2	9.08	Carbon dioxide ablative fractional resurfacing appears to be effective and well tolerated for the treatment of atrophic acne scars in Asians.
3	6.61	Fractional Er:YAG and CO_2_ lasers provided comparable outcomes of scar treatment, but fractional CO_2_ laser was associated with greater treatment discomfort.
4	5.79	Both NAF 1550 nm and AF CO_2_ lasers are effective in treating acne scars in ethnic skin with good patient satisfaction rate and high safety profile. PIH decreased with routine use of prophylactic bleaching creams. Fractional laser resurfacing open a wide horizon for treating acne scars in ethnic skin.
5	6.41	Treatment of facial acne scars with a diffractive lens array and 755‐nm picosecond laser produced improvement in appearance and texture at 3 months after the last treatment, with objective findings similar to those published for a series of fractional ablative laser treatments.
6	8.7	Topical PRP combined with FCL as an effective, safe modality in the treatment of atrophic acne scars with shorter downtime than FCL alone and better tolerability than FCL combined with ID PRP.
7	6.73	PRP has efficacy in the management of atrophic acne scars. It can be combined with microneedling to enhance the final clinical outcomes in comparison with microneedling alone.
8	5.92	Platelet‐rich plasma combined with fractional carbon dioxide laser treatment did not produce any statistically significant synergistic effects and also resulted in more severe side effects and longer downtime.
9	7.09	PRP treatment increased fibrogenetic molecules induced by fractional CO_2_ laser, which have association with clinical effect.
10	7.11	Both fractional 1064‐nm NdYAG picosecond laser and fractional 1550‐nm erbium fiber laser are safe and effective in the treatment of acne scars. Costs should be taken into consideration when deciding on which device to use to maximize treatment outcomes.
11	6.98	The combined use of fractional CO_2_ laser and PRP achieved better results. It reduced the downtime of the fractional CO_2_ laser. The use of the skin analysis camera provided an objective assessment of the results.
12	6.4	Fractional picosecond 1064‐nm laser was as effective as fractional carbon dioxide laser in treating atrophic acne scars, correlating with evidence of tissue remodeling with more safety profiles.

### 3.6 Analysis of Keywords

**FIGURE 8:**
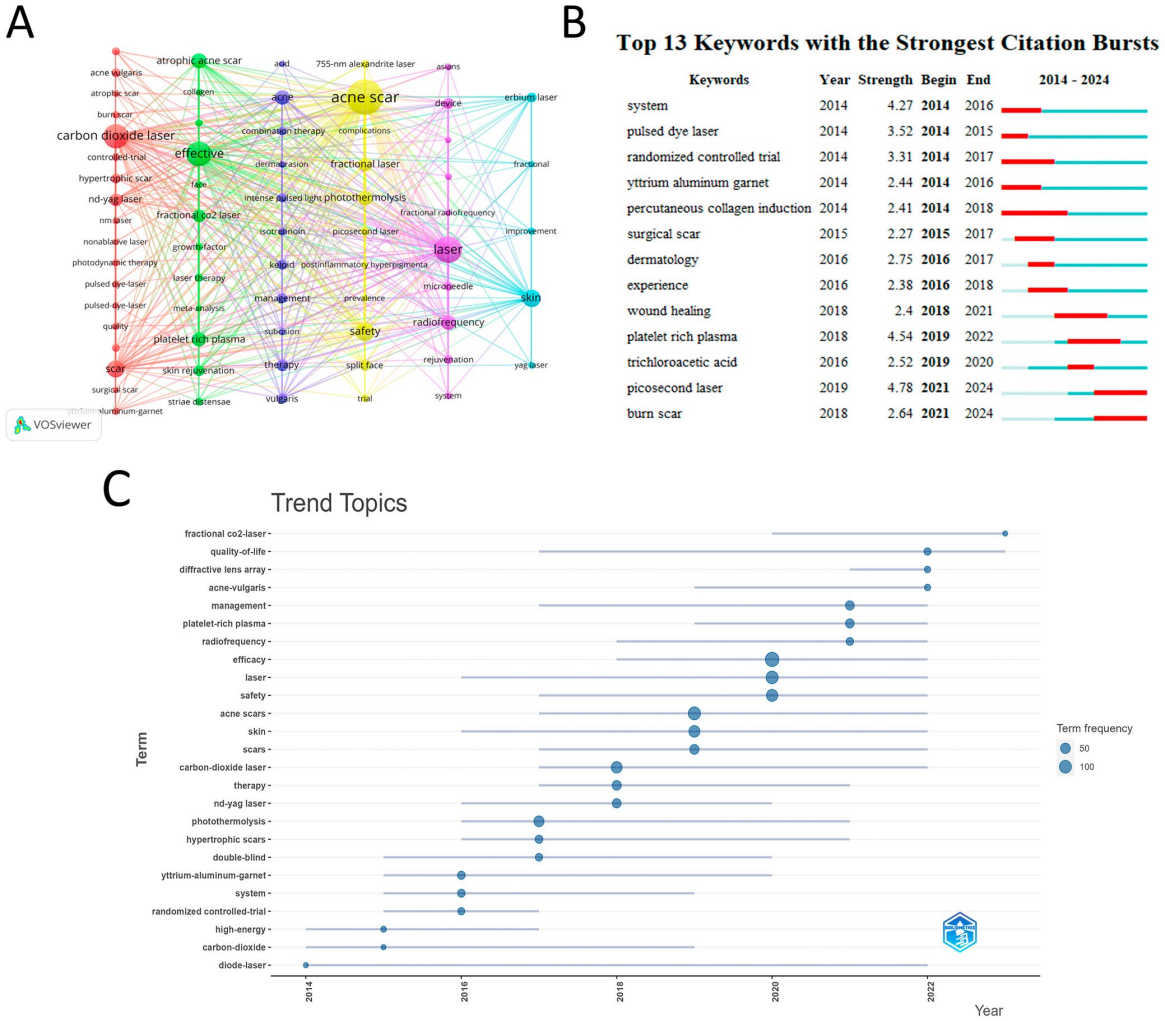
The keywords analysis. (A) The visual analysis of keywords in laser treatment for acne scars research field. (B) Keyword burst in Laser Treatment for acne Scars research field. (C) Annual trend chart of keywords changes.

Co‐occurrence analysis of keywords can effectively identify research hotspots in a specific field. Table 7 lists the top 20 high‐frequency keywords in research on laser treatment for acne scars. Excluding the search terms, “efficacy” appeared over 100 times, indicating its significance as a primary research hotspot in this field. We filtered keywords that appeared 15 times or more and performed a cluster analysis using VOSviewer (Figure 8A). The strength of connections between keywords is indicated by the thickness of the lines in the network.

**TABLE 7:** Top 20 keywords in laser treatment for acne scars research field.

Rank	Keywords	Counts	Rank	Keywords	Counts
1	Acne scar	262	11	Radiofrequency	60
2	Laser	164	12	Platelet rich plasma	57
3	Effective	146	13	Fractional laser	53
4	Carbon dioxide laser	141	14	Fractional CO_2_ laser	48
5	Safety	88	15	Nd:YAG laser	48
6	Skin	81	16	Therapy	42
7	Scar	79	17	Device	37
8	Atrophic acne scar	64	18	Split face	37
9	Acne	60	19	Skin rejuvenation	36
10	Photothermolysis	60	20	Keloid	35

Figure 8A displays six different clusters representing various research directions. The red cluster includes keywords such as the acne vulgaris, atrophic scar, burn scar, carbon dioxide laser, controlled trial, hypertrophic scar, Nd: YAG laser, nm laser, nonablative laser, photodynamic therapy, pulsed dye‐laser, quality, scar, surgical scar, yurlum–aluminum–garnet. The green cluster contains keywords like atrophic acne scar, collagen, face, fractional CO_2_ laser, effective, growth factor, laser therapy, meta‐analysis, skin rejuvenation, striae distensae, PRP. The blue cluster consists of keywords such as acid, acne, combination therapy, dermaorasion, intense pulsed light, isotretinoin, keloid, management, subcision, therapy, vulgaris. The yellow cluster contains keywords such as 755‐nm alexandrite laser, acne scar, complications, fractional laser, photothermolysis, picosecond laser, postinflammatory hyperpigmenta, prevalence, safety, spilt face, trail, and safety. The purple cluster includes keywords like Asians, device, fractional radiofrequency, laser, microneedle, radiofrequency, rejuvenation, system. Finally, the sky blue cluster contains keywords such as erbium laser, fractional, improvement, skin, YAG laser.

These clusters offer insights into diverse research directions in laser treatment for acne scars. Research on the efficacy of laser treatments for acne scars is a major focus in this field, with fractional CO_2_ laser being the most commonly utilized. Keyword co‐occurrence reveals emerging research areas, encompassing other laser types like the Nd: YAG laser and picosecond laser, along with combination therapies integrating fractional CO_2_ laser with radiofrequency and PRP.

Keyword burst analysis identifies the evolution of research hotspots by examining the temporal characteristics of keywords [19]. This aids researchers in comprehending future trends and directions in the field. Figure 8B illustrates the top 13 keywords with the highest burst strength from 2014 to 2024. The keyword with the highest burst strength is picosecond laser (4.78), followed by PRP (4.54), both of which also had longer burst durations (4th year). Moreover, the keywords that continued to exhibit bursts until 2024 are picosecond laser and burn scar, underscoring them as pioneering research domains in the field of laser treatment for acne scars.

The trend topic analysis of the keywords showed that from 2015 to 2021, the research during this period mainly focused on laser and scars types, and that the main keywords were carbon‐dioxide, photothermolysis, Nd:YAG laser, hypertrophic scars and acne scars, with a growing emphasis on the efficacy and safety of treatments (Figure 8C). Since 2019, the research focus has gradually transitioned toward other modalities such as PRP, radiofrequency, and diffractive lens arrays.

## 4 Discussion

### 4.1 General Information

The absence of literature on laser treatment for acne scars before 2014 suggests a dearth of research foundation during that period, with limited exploration of the relationship between laser treatment and acne scars. Since 2014, there has been a gradual increase in research in this field, with an average annual publication rate of 48 papers. This indicates that laser treatment is increasingly garnering scholars' attention in the treatment of acne scars.

In terms of publication volume and citation frequency by institutions and countries, the United States leads followed by China. Worth noting, despite China ranking second in both publications and citations, none of its institutions are in the top 10. This indicates that research institutions in this field are relatively dispersed in China, lacking deep involvement from any specific institution. In contrast, the United States has four institutions in the top 10, and these four institutions collaborate closely together. These institutions collaborate closely, indicating their leading position in this research field within the United States.

From the perspective of publishing journals, the journals in this field mainly fall within the fourth quartile in terms of quality and nature of research, with an average impact factor of 2.7. The Journal of Cosmetic Dermatology (IF = 2.3, Q4) has become the primary journal for publishing research in this field, indicating its current prominence. It is noteworthy that the American Journal of Clinical Dermatology (IF = 7.3, Q2) boasts the highest impact factor among the analyzed journals, yet it only has nine publications. This outcome suggests that laser treatment for acne scars has not garnered significant attention from researchers in terms of publication quantity, potentially indicating a lower quality and depth of research. This could be attributed to factors such as inadequate inclusion of study participants or a prevalence of case reports, resulting in a scarcity of high‐quality randomized controlled trials.

In our author analysis, Manuskiatti Woraphong from Mahidol University in Thailand emerges as the most prolific author in this field. He has closely collaborated with Wanitphakdeedecha Rungsima from Thammasat University in Thailand, jointly publishing 16 papers focusing on the safety and efficacy of microneedle radiofrequency, 1064 nm picosecond laser, and microlens array treatments for acne scars in Asian patients [20, 21, 22]. Kwon Hyuck‐Hoon, a scholar from South Korea, ranks third in terms of publications. His research mainly concentrates on the efficacy of 1550 nm nonablative erbium laser and microneedle radiofrequency, 1064 nm Ndpicosecond laser, Erlaser, and bipolar radiofrequency combined with infrared diode laser for treating acne scars [23, 24, 25]. Besides studying the effectiveness of different types of lasers for acne scars, these authors have also investigated the efficacy of CO_2_ fractional laser combined with adipose‐derived stem cell exosomes or PRP for treating acne scars. They found that the combination of exosomes or PRP increased clinical efficacy compared to using lasers alone [26, 27]. Overall, these researchers continue to focus on the efficacy and safety of various types of lasers alone or in combination for acne scars. However, the impact factors of their published articles are not high, potentially attributed to factors such as the number of patients included and single‐center studies. Therefore, strengthening collaboration among researchers from different countries and establishing mutually beneficial partnerships play a crucial role in the advancement of this field.

Keywords enable us to quickly identify the distribution and evolution of research topics in a field. By merging and counting keywords with similar meanings, we found that current research on laser treatment for acne scars mainly focuses on different types of lasers and their efficacy. Through CiteSpace analysis, we obtained the 13 keywords with the strongest citation bursts. Keywords with earlier citation bursts can reflect the topics that received early attention in the field. From Figure 8B, we can see that the early‐stage focus was on keywords such as system (2014–2016), pulsed dye laser (2014–2015), randomized controlled trial (2014–2017), yttrium aluminum garnet (2014–2016), and percutaneous collagen induction (2014–2018). In recent years, keywords such as PRP (2019–2022), trichloroacetic acid (2019–2020), picosecond laser (2021–2023), and burn scar (2021–2024) have gradually become research hotspots. Among them, the picosecond laser has the highest burst strength (4.78), indicating that it has become a focal point of research in the field of laser treatment for acne scars.

### 4.2 Hotspots and Frontiers

This study offers an overview of the current research status of laser treatment for acne scars. The results indicate that fractional CO_2_ laser has become the most widely used laser for treating acne scars, showing significant clinical efficacy. Nonetheless, clinical side effects such as erythema, pain, swelling, and postinflammatory hyperpigmentation still occur. Therefore, in recent years, researchers both domestically and internationally have increasingly combined fractional CO_2_ laser with other therapies to enhance the treatment of acne scars, aiming to mitigate the side effects of fractional laser while maintaining treatment safety and efficacy. Next, we will briefly outline some of the newer combination therapies.

### 4.3 Combining CO_2_ Laser With PRP

PRP is a plasma derived from autologous blood, rich in platelets and various growth factors, capable of activating and accelerating the cascade reaction of wound healing, thereby stimulating collagen synthesis and neovascularization [28, 29]. In recent years, PRP has emerged as an important clinical treatment modality, demonstrating efficacy in various dermatological conditions such as skin rejuvenation, hair loss, striae gravidarum, and acne scarring [30]. Recent studies have indicated that combining PRP with ablative fractional CO_2_ laser therapy is effective in treating atrophic acne scars [31]. For example, Aljefri et al. conducted a systematic review and meta‐analysis of 11 randomized controlled trials involving 313 patients with acne scars. The results demonstrated that the combined therapy of PRP and CO_2_ fractional laser significantly improved acne scars compared to laser therapy alone. Both clinical improvement and patient satisfaction were notably higher in the combination therapy group, with significant enhancements observed in the Goodman and Baron acne scar grading scale. Furthermore, the combined application of laser and PRP also reduced the duration of posttreatment erythema and edema. Based on these findings, the combination therapy of PRP and laser treatment may prove to be a novel approach for the treatment of severe atrophic acne scars.

### 4.4 Combining CO_2_ Laser With Stem Cells and Their Derivatives

Mesenchymal stem cells (MSCs) are undifferentiated, nonspecific cells in the human body with self‐renewal and multidirectional differentiation potential. They can differentiate into specific functional cells such as osteoblasts, chondrocytes, endothelial cells, and adipocytes, contributing to tissue regeneration and repair. This technology has been widely applied in tissue engineering and regenerative medicine research. Currently, two types of stem cells used for acne scar treatment are amniotic fluid‐derived mesenchymal stem cells (AF‐MSCs) and adipose‐derived stem cells (ADSCs). They repair necrotic and senescent cells and promote tissue regeneration. Stem cell therapy not only promotes the proliferation of dermal fibroblasts through direct cell‐to‐cell contact but also secretes various cytokines such as keratinocyte growth factor (KGF), vascular endothelial growth factor (VEGF), basic fibroblast growth factor (FGF), and platelet‐derived growth factor (PDGF) to regulate inflammation and exert anti‐fibrotic effects [32]. Therefore, in recent years, many scholars have applied stem cells and their derivatives to clinical and basic research on acne scars, finding that they can improve scar formation [33, 34, 35]. Zhou et al. [36] conducted a clinical study on 13 acne scar patients using CO_2_ laser combined with allogeneic ADSCs. One side of the face received laser treatment alone, while the other side underwent laser treatment combined with ADSCs. The results demonstrated a significant increase in skin elasticity and hydration, resulting in smoother and fairer skin in the combined treatment group. Histological analysis revealed an increased density of dermal collagen and elastic fibers. Due to the immunogenicity and incompatibility issues associated with stem cells, researchers in clinical settings often use conditioned medium (CM) for their studies. It has been found that fractional CO_2_ laser combined with adipose‐derived stem cell conditioned medium (ADSC‐CM) can also significantly improve acne scars [37]. In addition, some researchers have combined human amniotic fluid‐derived mesenchymal stem cells (AF‐MSCs‐CM) with microneedle therapy for acne scars and found that the improvement in scars in the combined group was also higher than that in the control group [38]. Furthermore, some scholars have used fractional laser combined with PRP or AF‐MSCs‐CM to treat acne scars and found that compared to laser treatment alone, combined therapy was more effective in improving acne scars, with no adverse reactions reported [39]. In summary, although there is currently limited clinical and basic research, which cannot definitively confirm the long‐term efficacy and specific mechanisms of stem cells and their derivatives in treating acne scars, they still hold therapeutic potential in the field of acne scar treatment. Therefore, they can be considered adjunctive treatment modalities to laser or microneedle therapy.

### 4.5 Combining CO_2_ Laser With Microneedle Radiofrequency

Microneedle radiofrequency (MNRF) is a minimally invasive treatment method that creates perforations in the epidermis. It delivers radiofrequency‐generated heat energy to the dermis through microneedles, inducing thermal injury, stimulating new collagen production, and minimizing damage to the epidermis, with minimal side effects. The mechanical effects of microneedles on the skin can improve transdermal absorption of topical products, promote growth factor secretion, and stimulate migration and proliferation of adjacent keratinocytes and fibroblasts, achieving skin remodeling [40]. Fractional laser therapy promotes wound healing and stimulates collagen and dermal remodeling by acting on the microthermal treatment zone, making it one of the gold standards for acne scar treatment. However, it is associated with long recovery times and noticeable side effects. Therefore, researchers have been combining the two methods in recent years to enhance treatment efficacy while minimizing risks and shortening treatment duration [41]. Canpolat et al. [42] conducted a self‐controlled study involving 41 acne scar patients treated with CO_2_ fractional laser combined with MNRF. The results showed that compared to before treatment, combination therapy significantly reduced side effects while effectively improving acne scars. Similarly, Tatlıparmak et al. [43] conducted a study involving 71 patients treated with CO_2_ fractional laser combined with MNRF, resulting in significantly improved patient satisfaction and a notable decrease in the clinical evaluation of acne scar severity using the ECCA scoring system.

### 4.6 Combining CO_2_ Laser With Medications

In recent years, to address issues such as erythema, pigmentation, and compromised skin barrier after CO_2_ fractional laser treatment of acne scars, the combination of Centella ointment, polysulfated glycosaminoglycan cream, growth factors, and hyaluronic acid has emerged as a new approach in clinical acne scar treatment. In a randomized controlled trial with 20 patients having acne scars, one side of the face was treated with a fractional CO_2_ laser combined with Centella ointment, while the other side was treated with the laser combined with a steroid cream. The study found no significant differences between the two treatments in overall efficacy, average downtime, incidence of adverse reactions, and wound healing improvement. However, the side treated with the laser and Centella ointment showed a lower incidence of postinflammatory hyperpigmentation. This confirms that combining a fractional CO_2_ laser with Centella ointment can enhance treatment efficacy, reduce adverse reactions, and shorten recovery time after laser treatment. These benefits may be attributed to Centella ointment's promotion of granulation tissue formation and wound healing [44]. Similar results were obtained by Damkerngsuntorn et al. [45] through combining Er:YAG laser with extract of *Centella asiatica*. Additionally, due to the reparative effects of bovine basic fibroblast growth factor (bFGF) on the skin barrier and wound healing, some researchers have combined it with CO_2_ fractional laser treatment for acne scars, finding that the combination therapy aids in repairing the compromised skin barrier post‐laser treatment [46]. Moreover, pigmentation is a significant adverse reaction following laser treatment of acne scars, and polysulfated glycosaminoglycan is believed to improve pigmentation and promote wound healing and skin repair. Therefore, researchers have found that combining fractional laser treatment with topical polysulfated glycosaminoglycan cream accelerates wound tissue recovery, thereby facilitating the formation of new skin tissue and ultimately diminishing scar appearance. In addition to the aforementioned study, hyaluronic acid gel dressings, known for their moisturizing properties and ability to accelerate tissue wound healing, have been investigated in recent years. Researchers have explored their use in mitigating adverse reactions following fractional CO_2_ laser treatment for acne scars. Studies, such as that conducted by Zhang et al. [47] have confirmed that combining hyaluronic acid with CO_2_ fractional laser treatment for acne scars not only further enhances clinical efficacy but also effectively improves ECCA scores and increases patient satisfaction.

In conclusion, the aforementioned studies suggest that irrespective of the combination method employed, both mitigating the adverse effects of laser treatment and achieving substantial therapeutic benefits are feasible in acne scar management. Nonetheless, specific evaluations of the efficacy of the aforementioned combination treatments are presently absent. Therefore, future multicenter collaborative research is required to determine the optimal treatment approach for acne scars.

### 4.7 Limitations

This study also has certain limitations. Firstly, the data are exclusively from the WoSCC database, potentially overlooking relevant studies from other databases. Secondly, the inclusion of solely English literature introduces language bias. Additionally, recent high‐quality literature may have low citation frequencies due to short publication times, possibly causing discrepancies between analyzed research results and the actual scenario. Lastly, our bibliometric analysis method gathers only general information from articles, neglecting nuanced content such as authors' viewpoints and forward‐looking opinions. Moreover, we only summarize high‐frequency terms in data analysis sections like keywords, potentially missing insights into current research trends like stem cell research.

## 5 Conclusion

In summary, due to the continuous growth in the number of articles on laser treatment for acne scars over the past decade, accurately and rapidly analyzing research in this field and describing its trends have become particularly crucial. In this study, we employed software such as VOSviewer, CiteSpace, Excel, and R package to analyze trends and hotspots in laser treatment for acne scars from 2014 to 2024. We summarized and analyzed various aspects including countries, journals, research institutions, authors, and keywords. Our findings indicate that the United States and China are leading in this field, with Mahidol University in Thailand and Cairo University in Egypt being core institutions. The Journal of Cosmetic Dermatology has the highest number of publications in this field, with most journals being in the fourth quartile with relatively low impact factors. There is a lack of cooperation and communication between some countries and institutions, especially in China. Therefore, improving research quality and publishing high‐quality clinical studies should be a concern for clinicians. Laser combined with PRP, microneedle radiofrequency, stem cells, and drugs has gradually become a hotspot and trend in this research field. It is hoped that this study can provide a reference for domestic researchers.

## Author Contributions

Yang Wen was responsible for the conceptualization, methodology, formal analysis, and writing of the original draft. Yuan Cai handled data curation, software development, visualization, formal analysis. Lanfang Zhang contributed to data collection, resources, and visualization. Lin Li was involved in data curation, statistical analysis, and validation. Jing Wang assisted with literature search, methodology, and validation. Feng Jiang supervised the study, contributed to writing review and editing. Nana Sun provided supervision, conceptualization, review and editing, and validation. Ni Zeng contributed to the conceptualization, review and editing, and managed the project. All authors read and approved the final manuscript.

## Ethics Statement

The authors have nothing to report.

## Consent

The authors have nothing to report.

## Conflicts of Interest

The authors declare no conflicts of interest.

## Data Availability

The data that support the findings of this study are available from the corresponding author upon reasonable request.
